# Atlanta Society of Medicine

**Published:** 1898-06

**Authors:** L. A. Felder

**Affiliations:** Secretary; Atlanta, Ga.


### SOCIETY REPORTS.

### ATLANTA SOCIETY OF MEDICINE.

Atlanta, Ga., April 7, 1898.

The Society was called to order by the President at 8 o'clock P. M., and on call of the roll by the secretary, the following members answered: Drs. Amster, Blalock, Campbell, J. L., Champion, Childs, Divine, Duncan, Goldsmith, Hancock, Hutchins, Kendrick, McDaniel, Smith, C. E. J., Visanska, and Felder.

*The President:* I am glad to be able to state, gentlemen, that we have with us to-night Dr. George H. Fox, of New York, who will give us an address on "Syphilitic Eruptions," and demonstrate his lecture with stereopticon pictures.

*Dr. Fox:* Allow me to express the pleasure it has given me of being to-day, for the first time in my life, in the city of Atlanta. I have enjoyed your busy streets, fine buildings and delightful weather, and that one attraction which we certainly cannot equal in New York, the delightful winding bicycle track out on the outskirts, and that delightful hospitality which I cannot say is peculiar to Atlanta, as I have often heard it spoken of as abounding in all Southern cities.

I am here to-night, not with an original address, but rather in the capacity of a showman, and the pictures I will show will speak probably more eloquently and certainly more instructively than I could.

The science of dermatology, like botany, is considered very difficult to acquire. These must be learned by a careful study of the cases themselves, because, although well prepared paintings may be of great service in learning it, we must learn to recognize certain traits that are peculiar to different species. We can distinguish a maple from an elm, and perhaps some of you could distinguish a chestnut from a hickory, though I doubt if I could, because we have seen trees of these kinds again and again and can recognize perhaps instinctively the individual characteristics of them. Yet if we were asked a quarter of a mile off why we knew a certain tree to be an elm, perhaps it would be hard for us to define the reason. In the same way we recognize the nationality of men; we say that this one is an Irishman, another a Frenchman, another a German. Perhaps we cannot say exactly why we know this, but we have trained ourselves to unconsciously observe certain peculiarities, certain characteristics, which tell at once the nationality. So it is with diseases of the skin. Each one is marked with certain peculiarities which are very perceptible whenever we have a sufficient amount of experience to distinguish them. I am glad to say that syphilitic eruptions are so peculiar that in nearly every case they tell what they are as plainly as if it were written on the skin. It is not often necessary to ask a man whether he ever had a chancre or ever had syphilis or not. The characteristics of syphilitic eruption are often so plainly shown on the skin that you can recognize it at the first glance, and certainly after a careful study, except in very rare cases where it is difficult to distinguish certain forms of it from certain forms of psoriasis; and I have seen occasional special cases that have been modified in appearance by treatment where the diagnosis is not easily made. But the point I wish to make is this: The diagnosis can be made better by what we see than by what the patient tells us. The man who begins his diagnosis by asking questions is certain in many cases to be led off the track. We must remember that there are some patients who have had syphilis and have something else. The man who has had syphilis is not exempt from psoriasis or a multitude of other things. On the other hand you will find a man who has a well-marked syphilitic eruption who will tell you that he has never been exposed. You have often heard the remark that any syphilitic patient will lie. Now I don't believe this generally, but there are many men who have had an initial lesion and have been unaware of it, or have forgotten it—have never been treated—ten or twenty years ago; they have a plainly marked syphilitic eruption, and yet if the physician depends upon the patients he will certainly be misled.

In a vast majority of the cases the diagnosis is written on the skin so plainly that he who runs may read. Now this is not always true, though nearly always. In a case of locomotor ataxia you must depend upon the history of the case. So you cannot distinguish perhaps between iritis the result of syphilis and iritis the result of some other cause. But syphilitic skin diseases have certain peculiarities which tell distinctly what they are, and it is some of these peculiarities that I wish to present to you to-night, in one case after another, so that they will be impressed upon your minds.

Dr. Fox then proceeded with the exhibition of the stereopticon views, explaining each one as it was presented.

*Mr. President:* You have heard this interesting talk by Dr. Fox. We would like to hear it discussed. Dr. Hutchins, we would like to hear from you.

*Dr. Hutchins:* I have long had an idea, and I think a correct one, that the majority of eruptions cannot be pictured, and that in pictures, whether colored or not, skin diseases, or skin manifestations of disease, if you take the label off or take away the man who knows what they are to tell you, you cannot make a diagnosis from the pictures. But I am sure that Dr. Fox's illustrations, photographs, come nearer enabling us to make a diagnosis than any pictures I have ever seen. Of course in some of the pictures you can get nothing but the configuration and distribution of the disease; yet in the great majority of these pictures you can tell from the arrangement and grouping that it is syphilitic. In some of the others of course you cannot.

There is nothing to say except that Dr. Fox has shown that he is an expert photographer as well as good in his diagnostic work, and I feel as much obliged to him as anybody could for these pictures. As regards his treatment, he follows rather broad lines. I am inclined to think he is right. There are cases of chronic malaria that cannot be cured with quinine, and there are many other like cases. If you can get the patient up to where he is in proper condition it will do more than the drugs. Sometimes the best thing you can do for a syphilitic patient is to stop the treatment entirely, give them no treatment and put them on good diet, or give them an absolute change.

*The President:* Is there any other miscellaneous business before the Society?

*Dr. Amster:* I have a communication here from Dr. Howard A. Kelly, of Baltimore. He writes regarding some pernicious legislation which the anti-vivisection society is trying to pass in Washington. I would like for you to have this read before the Society, and if the Society thinks proper communicate with our senators and ask them not to support any measure of that kind.

The Secretary then read Dr. Kelly's letter to the Society.

*The President:* You have heard this letter, gentlemen. What shall we do with it, if you desire to take any action in regard to it?

*Dr. Amster:* Not having any personal acquaintance with either of our senators, I thought this would be the proper channel for it to go through, so that if any of the gentlemen here personally know Senator Clay or Senator Bacon they can communicate with them.

Upon motion the following resolution was adopted, and the Secretary was instructed to forward copies of the same to each senator and representative in Congress from Georgia:

"*Resolved,* That the Atlanta Society of Medicine, being opposed to the passage of the anti-vivisection bill now pending before Congress, as being a measure calculated to do great harm to the progress of the science and practice of medicine, and consequently be of great damage to the people of the United States and of the world; the senators and members of Congress from Georgia are respectfully asked to use their efforts towards the defeat of the bill."

L. A. FELDER, M.D., *Secretary.*

---

"Biliousness" is a contraindication to the exhibition of iron· "So long as there is foul tongue, a bad taste in the mouth (as if it could be any place else), and fullness of the liver, with disturbance of the alimentary canal, iron is to be prohibited; it is not only that it is of no service, it positively does harm."—*Medical Age.*

